# Arterial stiffness adaptations to chronic resistance and aerobic exercise: a systematic review of exercise modalities

**DOI:** 10.3389/fpubh.2025.1701763

**Published:** 2026-01-15

**Authors:** Ina Shaw, Musa L. Mathunjwa, Jane Black, Razieh Khanmohammadi, Takalani Clearance Muluvhu, Gregory A. Brown, Gudani G. Mukoma, Brandon S. Shaw

**Affiliations:** 1 School of Sport, Rehabilitation and Exercise Sciences, University of Essex, Colchester, United Kingdom; 2 Division of Public Health, University of the Free State, Bloemfontein, South Africa; 3 Department of Human Movement Science, University of Zululand, KwaDlangezwa, South Africa; 4 School of Health and Sports Sciences, University of Suffolk, Ipswich, United Kingdom; 5 Department of Sports Science, Urmia University, Urmia, Iran; 6 Department of Sport, Rehabilitation and Dental Sciences, Tshwane University of Technology, Pretoria, South Africa; 7 Physical Activity and Wellness Laboratory, Department of Kinesiology and Sports Science, University of Nebraska at Kearney, Kearney, NE, United States; 8 Department of Biokinetics, Recreation and Sport Science, Faculty of Health Sciences, University of Venda, Thohoyandou, South Africa; 9 SAMRC Wits Developmental Pathways for Health Research Unit (DPHRU), School of Clinical Medicine, Faculty of Health Sciences, University of the Witwatersrand, Johannesburg, South Africa

**Keywords:** older adult(s), endothelium, exercise, hemodynamics, pulse wave velocity, vascular stiffness

## Abstract

**Background:**

Arterial stiffness is a strong predictor of cardiovascular morbidity and mortality, especially in older adults. Despite exercise being shown to positively influence arterial health, the relative effectiveness of various exercise modalities remains unclear.

**Objectives:**

This systematic review aimed to examine the effects of resistance training (RT), aerobic training (AER), and concurrent aerobic plus resistance training (CON) on arterial stiffness in older adults, a recognised modifiable and independent cardiovascular risk factor.

**Methods:**

A comprehensive search of Medline (EBSCO), EMBASE, the Cochrane Database of Systematic Reviews, and clinicaltrials.gov was conducted. The robust search strategy, which also included systematic reviews being hand-searched for relevant articles and a forward and backwards citation search on the included articles, was employed to reduce the risk of selection and publication biases. Studies included in the review assessed arterial stiffness using pulse wave velocity (PWV) and focused on individuals aged 60 years and above who participated in a chronic (≥ 8 weeks) structured exercise intervention (RT, AER, or CON). Only randomised controlled trials were included. Sixteen studies met the inclusion criteria, and their methodological quality was evaluated using the PEDro scale.

**Results:**

The findings indicated that resistance training generally had a neutral effect (mean difference, MD: 0.06 m/s. SD: ±0.45) on arterial stiffness, whilst aerobic training produced modest improvements (MD: −0.62 m/s, SD: ±0.51). Notably, concurrent training consistently reduced arterial stiffness across diverse older adult(s) populations (MD: −0.85 m/s, SD: ±0.63).

**Conclusion:**

Combined aerobic and resistance training is the most effective non-pharmacological strategy for reducing arterial stiffness in older adults. This approach may offer essential benefits for cardiovascular health and healthy ageing. Further long-term studies are needed to explore the mechanisms involved and to inform tailored exercise interventions in geriatric populations.

## 1 Introduction

Cardiovascular diseases are a leading cause of death globally, accounting for an estimated 38% of the 17 million premature deaths (under 70 years of age) (1). Understanding age-related cardiovascular changes is essential for the optimal prevention and management of cardiovascular disease (CVD) among older adults (2). Ageing is associated with significant changes to the heart and vascular structure and function, which makes older adults more susceptible to CVDs and resultant morbidity and mortality (3). Specifically relating to the vascular system, ageing results in significant structural remodelling and functional changes because of calcification, increased arterial wall thickness/diameter, loss of elasticity, increased collagen deposition, elastin fragmentation in the media layer and/or an impaired endothelial vasoreactivity (4). All these factors make the vessels stiffer and less elastic or resilient. This physical stiffening of the larger central arterial system forms the central model of vascular ageing (5).

Arterial stiffness increases progressively with age, this physio pathogenesis of vascular ageing is associated with the pathogenesis of a large spectrum of CVDs, such as systolic hypertension, coronary artery disease, cerebrovascular accidents, heart failure and atrial fibrillation, and non-cardiovascular outcomes, such as cerebral white matter lesions and several types of cognitive deficits (6), which are the leading causes of mortality worldwide. As such, increasing arterial stiffness has become a powerful and independent predictor of CVD or adverse cardiovascular outcomes in asymptomatic individuals (7).

Arterial stiffness is increasingly recognised as an urgent and modifiable CVD risk factor in the ageing population (8, 9). It is a key predictor of adverse cardiovascular events such as hypertension, stroke, and heart failure, conditions that disproportionately affect the older adult(s) (10). With the global ageing population on the rise, the prevalence of arterial stiffness continues to grow, significantly contributing to morbidity and mortality rates (11). Despite its critical impact on health outcomes, effective interventions to reduce arterial stiffness are still under-researched, making it a pressing public health concern (12). Addressing this issue through targeted interventions offers a promising non-pharmacological approach to mitigate cardiovascular risks and improve the quality of life in older adults. In this regard, since age-related changes in arterial stiffness are associated with differences in smoking habits, cholesterol levels, nutritional characteristics, blood glucose levels and regular exercise, the development of novel therapeutic strategies to impact these modifiable risk factors to reverse this process has begun to gain increasing attention (13). Over many decades of research in cardiovascular ageing, the role of regular exercise has become increasingly apparent in attenuating age-related decline in cardiovascular function (13). In this vein, recent studies have also demonstrated that the physiopathogenesis of vascular ageing can be slowed, reduced, or even reversed by regular exercise (14).

Whilst all mechanisms through which exercise affects arterial stiffness are not yet understood, it may do so through several physiological mechanisms. One primary mechanism is the improvement in endothelial function, where regular exercise enhances the release of nitric oxide (15), a vasodilator that helps maintain vessel flexibility (16). Regular exercise also affects the structural components of the arteries, such as elastin and collagen, which play key roles in arterial flexibility. Over time, regular physical activity can lead to favourable changes in these proteins, resulting in more elastic arteries (17). Additionally, exercise may reduce chronic inflammation and oxidative stress, and improve antioxidant capacity, all of which contribute to arterial stiffening (18, 19). Long-term regular exercise also improves atherosclerosis characteristics, such as improved serum lipid profiles, enhanced reverse cholesterol transport, and reduced atherosclerotic plaque (20). Whilst aerobic exercise has been shown to reduce sympathetic nervous system activity, leading to lower vascular resistance and improved arterial compliance (21), resistance training may contribute by increasing muscle strength, which can indirectly improve arterial stiffness through better blood pressure regulation (22). Combining these two exercise modalities may prove superior to any one modality in isolation. Crucially, a comparative analysis across sex and major racial/ethnic groups is needed to determine the generalizability of these training effects.

Further support for the role of exercise training in preventing and reducing high arterial stiffness is becoming evident from analysis indicating lower arterial stiffness in individuals with a higher aerobic capacity (14). In addition, whilst most studies have focused on the role of aerobic training on arterial stiffness (20, 23), some studies have investigated the differences between aerobic training and other exercise training types (24, 25), albeit in younger adults. When research is forthcoming on the long-term role of aerobic training on arterial stiffness in younger adults, it has been conflicting. This is especially true regarding the roles of resistance training (RT) alone or in combination with aerobic training on arterial stiffness. This is problematic because the effect on arterial stiffness may be varied in response to different exercise modalities due to underlying differences in the molecular mechanisms underlying the difference in the training effect (26). Therefore, the objective of this study was to systematically review the impact of RT or aerobic training alone versus concurrent aerobic plus resistance training on arterial stiffness responses in older adults.

## 2 Materials and methods

### 2.1 Eligibility and exclusion criteria

Only randomised controlled trials (RCTs) conducted in humans and published in English were considered for this study. In addition, the following criteria also had to be met to be included in the analysis: studies needed to include either a period of aerobic, Resistance Training (RT), and/or a combination of the two; arterial stiffness measured by Pulse Wave Velocity (PWV) (m/s) had to be evaluated at pre- and post-training intervention; the exercise intervention duration had to be at least 2 weeks; aerobic training and RT compared with other treatments, no treatment, health education, physical therapy, conventional medical treatment, compared to the same treatments were included; the studies must have had control groups for comparing different interventions; and participants in the studies had to be aged 65 years or older (27).

The exclusion criteria included: a different exercise modality; different population (i.e., participants with existing disease conditions); inappropriate study design (non-intervention RCT studies, theses, meta-analyses and systematic reviews); use of indirect measures of arterial stiffness (i.e., augmentation index); no full text on the listed electronic databases for published studies; and studies that also used another intervention (e.g., diet or nutritional supplements), other than aerobic or RT, in combination that may have impacted on arterial stiffness.

### 2.2 Search strategy and study selection

The following databases: Medline (EBSCO), EMBASE, Cochrane Database of Systematic Reviews and clinicaltrials.gov were searched for eligible studies. The search was conducted between July 2021 and March 2022. Search included articles from the database’s inception up to March 2022. Systematic reviews were hand-searched for relevant articles. A forward and backwards citation search was conducted on the included articles. The Medical Subject Headings (MeSH) terms and key words included were (1) arterial stiffness OR vascular stiffness OR Pulse wave velocity OR PWV OR baPWV OR brachial-ankle pulse wave velocity OR cfPWV OR carotid-femoral pulse wave velocity AND; (2) aged OR geriatric OR old age OR older OR older adult(s) OR older adult AND (3) resistance exercise OR resistance training OR strength exercise OR strength training OR weight training; OR (4) aerobic exercise OR aerobic training; OR (5) combination training OR concurrent training. Additionally, references of all selected articles and relevant systematic reviews were checked to identify other eligible studies.

The first and last authors independently conducted the title, abstract and full-text screening. Irrelevant and duplicate studies were removed by reviewing the titles and abstracts. All articles or RCT data with potentially relevant trials were downloaded and reviewed before final inclusion. Disagreements were resolved through discussion. When the same data were presented in multiple publications, the first published study was used for the analysis.

The quality of methods reported in each included study was evaluated through consensus of the first and last authors, using the PEDro Scale (28). Since participants, therapists, or assessors can rarely be blinded, items 4–6 were removed from the scale (29). A PEDro score of at least four was required for inclusion into this study to limit analytical bias of this review. The comprehensive search strategy, study screening, and quality assessment (PEDro score of age for inclusion) were performed independently by the first and last authors and confirmed through consensus of the entire author group to ensure methodological rigor and limit analytical bias.

### 2.3 Data extraction and synthesis

The methodology focused on extracting five key areas from included studies: authors and publication year; study population characteristics (age, health status, sample size); the detailed exercise program description (training type, duration, frequency, intensity, volume); the specific arterial stiffness measurement; and the primary exercise effects on arterial stiffness. These data points were narratively summarised due to heterogeneity.

**Figure 1:**
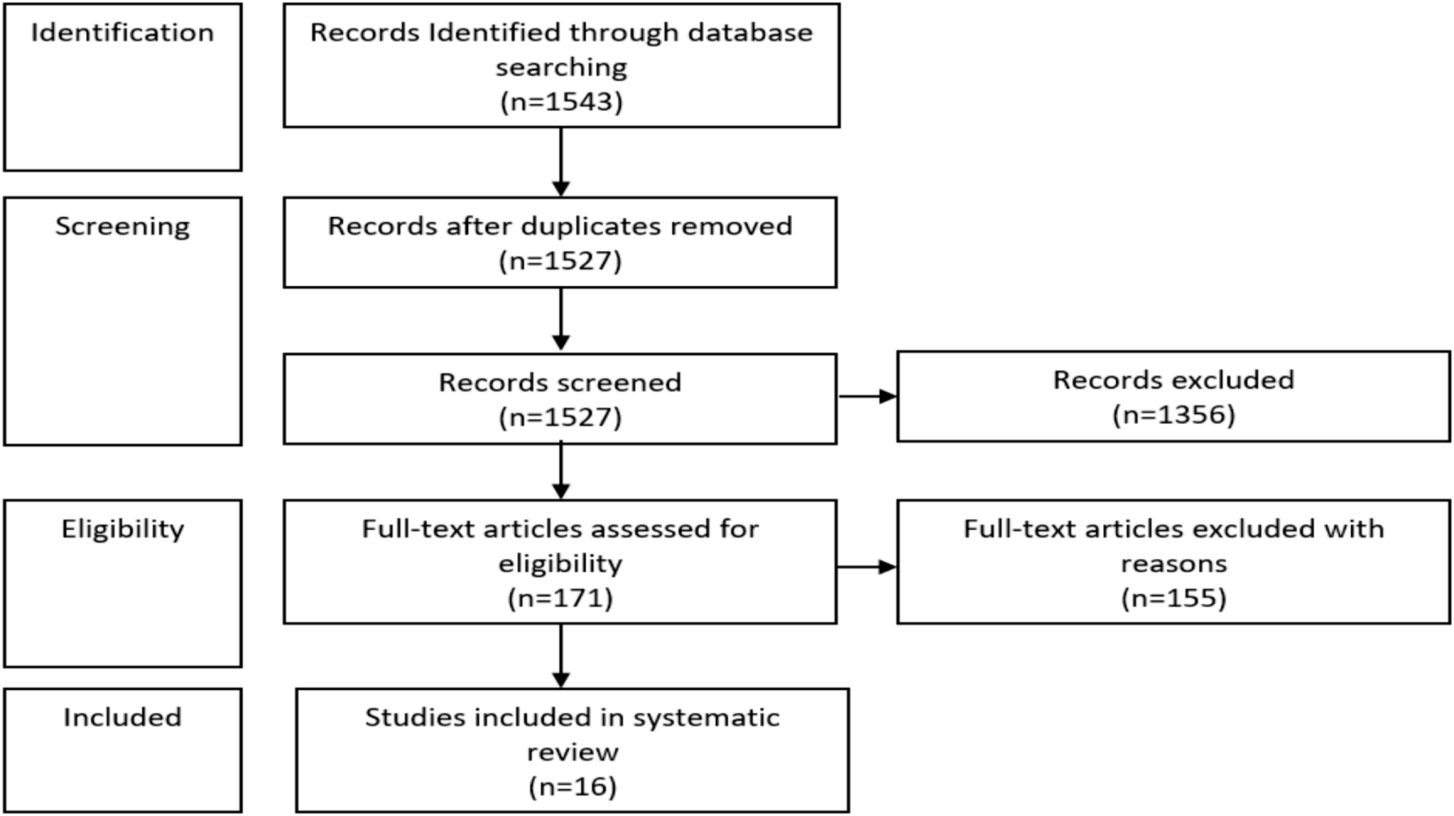
Flowchart of the study identification process.

Statistical Analysis: Due to significant heterogeneity observed across the included studies regarding participant populations, exercise protocols, and arterial stiffness measurement techniques, a formal quantitative meta-analysis and forest plot were not performed. Instead, a narrative synthesis was applied, and all individual study numerical outcomes, including mean change and variability, are detailed in Table 1. This review was not registered, and a protocol was not prepared. The present study made use of the PRISMA statement is an evidence-based minimum set of items, which includes a 27-item checklist and a four-phase flow diagram (Figure 1).

**Table 1:** Summary of included study outcomes: effect of exercise modalities on arterial stiffness (PWV) in older adults.

Study ID (author, year)	Intervention (modality)	*N* (Group/Total)	AS Measure	Pre-Training PWV (m/s) (SD)	Post-Training PWV (m/s) (SD)	Mean Change (*Δ*) (m/s)	Variability (95% CI or SD)	Significance (*p* < 0.05)
Lopes et al. (57)	RT (3x/wk., 12 wks)	*N* = 15	cfPWV	10.5 (1.2)	10.4(1.1)	−0.1	[−1.58, 0.42]	No
Jurik et al. (58)	AER (2x/wk., 24 wks)	*N* = 40	baPWV	16.2 (1.9)	16.5(1.8)	+0.3	[−0.2, 0.8]	No
Zhang et al. (59)	RT (3x/wk., 12 wks)	*N* = 15	cfPWV	10.5 (1.2)	10.4(1.1)	−0.1	0.35	No
da Silva et al. (60)	CON (4x/wk., 12 wks)	*N* = 50	baPWV	15.0 (2.1)	14.5 (2.1)	−0.5	0.45	No
Wainman, (46)	RT (3x/wk., 8 wks)	*N* = 24	cfPWV	9.0 (1.0)	9.2(1.0)	+0.1	0.28	No
Werner et al. (47)	AER (3x/wk., 12 wks)	*N* = 28	cfPWV	12.5 (1.3)	11.5(1.2)	−1.0	[−1.6–0.4]	No

RT, Resistance Training; AER, Aerobic Training; CON, Concurrent Training; AS Measure, Arterial Stiffness Measurement; cfPWV, Carotid-Femoral Pulse Wave Velocity; baPWV, Brachial-Ankle Pulse Wave Velocity; PWV, Pulse Wave Velocity. SD, Standard Deviation; CI, Confidence Interval.

## 3 Results

In all, 1,543 articles were identified in the search, of which 1,527 were excluded, with 16 articles being included in this study. Specifically, 965 studies were excluded after reading abstracts and titles, a further 391 articles did not meet the inclusion criteria for outcome variables and 16 duplicates were removed. The remaining 171 full-text articles were read and assessed for eligibility. Then, 155 studies were excluded for the following reasons: the RT and/or aerobic training was combined with other activities (i.e., blood flow restriction); the experimental group consumed supplements or medications along with exercise without a defined control group; arterial stiffness was measured by another method than PWV; the participants’ mean age was less than 65 years; the experimental group consumed supplements or medications along with exercise; studies utilised acute interventions or interventions lasting less than 2 weeks. Finally, 16 studies were included in the review with a mean PEDro scale score of 4.8. The evidence available was rated for all reported outcomes as moderate-certainty evidence, which means that we have confidence in the estimates of effect. Three interventions involved RT alone (30–32), six aerobic training alone (33–37) and seven concurrent training (38–44) and their role on arterial stiffness in the older adult(s). No studies compared RT to aerobic training, RT to concurrent training or aerobic training to concurrent training.

Altogether, these studies evaluated 1,102 participants. Two studies enrolled only men (41, 43), six studies enrolled only women (35, 38–40, 42, 44) and eight enrolled both men and women (30–33, 36, 37, 45) Specifically, 100% of the RT studies enrolled men and women (30–33), 83% of aerobic studies enrolled men and women (33–37) with 17% enrolling only women (35). In turn, 71% of the concurrent studies enrolled only women (38–44), whilst 29% enrolled only men (41, 43).

### 3.1 Effect of interventions

Detailed numerical outcomes, including pre- and post-intervention PWV values, the mean change, and measures of variability (95% CI or SD of the change score), for all included studies are presented in Table 1.

All (100%) of the sampled RT intervention studies failed to significantly influence arterial stiffness. The RT-only interventions ranged from 3 (32) to 5 months (30), and involved training two (31, 32) to three times weekly (30) using six (32) to eight repetitions (30) at an intensity ranging from 70% one-repetition maximum (1-RM) (30) to fatigue (31, 32).

Two-thirds (67%) of the sampled aerobic training studies reported decreased arterial stiffness (33–35), whilst 33% reported no significant effect on arterial stiffness (36, 37). Of the aerobic-only studies that report decreased arterial stiffness, training regimes ranged from 12 weeks (35, 37) to 6 months (34), with aerobic training performed two (34) to four times weekly (33) using bench stepping (35), walking (33, 34), running (34) and/or cycling (34). Those aerobic training interventions that failed to improve arterial stiffness used programs of 12 weeks (37)/3 months (36), performing cycling (36) or arm ergometry (37) three times daily (37) or three times weekly for 2035 to 30 min 34 (Table 1).

In turn, 100% of the concurrent studies decreased arterial stiffness (38–44). The sampled concurrent-only training studies ranged from 10 (43) to 52 weeks (42) performed one (39) to three (38, 41, 42, 45) times per week comprising of RT using four (38) to eight exercises (39), using three (39, 42, 43) to five sets (39, 40), and eight (43) to 20 repetitions (40) when combined with simple aerobic exercises of stepping 36, walking 38–40, 42, running 38–40 and/or cycling (39, 42, 43) at 40% heart rate reserve (HRR) (44) to 80% of age-predicted maximal heart rate (HR) (38) (Table 1).

## 4 Discussion

This study provides one of the few direct comparisons of chronic resistance, aerobic, and concurrent training on arterial stiffness in older adults, and is the largest to date. The results indicate that RT alone does not significantly influence arterial stiffness, whilst aerobic training leads to modest improvements. In contrast, concurrent training consistently demonstrates the greatest benefits. These findings suggest that integrating aerobic and resistance training may be the most effective approach for enhancing vascular health in older adults (see Table 2).

**Table 2:** Arterial stiffness responses to resistance, aerobic and/or concurrent aerobic plus resistance training in the older adult(s).

Author(s)	Characteristics of the study population and sample size	Description of the exercise program	Effect of exercise on arterial stiffness
Resistance training			
Manojlović et al. (30)	Overweight and obese older adults 68 ± 3 years *n* = 32	5 months 3 times per week 8 exercises 70% 1-RM	RTG: baPWV unchanged RT + Calorie restriction G: unchanged
Jefferson et al. (31)	Men and women from Northwest England 68.73 ± 5.80 years *n* = 100	16 weeks 2 times per week 7 exercises 2 sets <12 repetitions To fatigue for each exercise	RTG: PWV unchanged (+1.04 m/s) NonG: PWV unchanged (+1.00 m/s)
Kirk et al. (32)	Older adult(s) men and women 65 years *n* = 70	3 months 2 times per week (Tue & Fri) 6 exercises 1st circuit: 15 repetitions 2nd circuit: 12 repetitions 3rd & 4th circuit: 10 repetitions Repetitions and intensity to achieve significant fatigue	RTG: PWVao unchanged (10.34 ± 1 vs. 10.54 ± 2 m/s) Sitting callisthenic balance group: PWVao decreased (9.9 vs. 9.6 m/s)
Aerobic training			
Kujawski et al. (33)	Older men and women with obesity 69.2 ± 3.5 years *n* = 160	20 weeks 4 times per week Walking on a treadmill 15–30 min 50–75% of HRR EX+Mod-CR group after the intervention [compared with both the EX-only (*p* = 0.008; m = 3) and EX+High-CR groups]	AerG: Aortic arch PWV decreased [8.5 m/s (95% CI, 7.8–9.2)] EX+Mod-CR: Aortic arch PWV decreased [7.3 m/s (95% CI, 6.7–7.9)] EX+High-CR: Aortic arch PWV decreased [8.5 m/s (95% CI, 7.8–9.2)]
Brinkley et al. (34)	Older men and women with Type 2 Diabetes, Hypertension, and Hypercholesterolemia 71.4 ± 0.7 years *n* = 36	3 months 3 times daily 60 min Cycling and walking/running on treadmill 60–75% HRR	AerG: Radial PWV decreased (−20.7 ± 6.3%) Femoral PWV decreased (−13.9 ± 6.7%) NonG: Radial PWV unchanged (+8.5 ± 6.6%) Femoral PWV unchanged (+4.4 ± 3.3%)
Son et al. (45)	2 older adults with diet/oral hypoglycemic-controlled T2DM, hypertension and hypercholesterolemia 69.3 ± 0.6 years *n* = 72	6 months 3 times per week 60 min Cycling and walking/running on treadmill 60–75% of HRR	AerG: Radial PWV decreased at 3 months (−21.7 ± 6.7%) Radial PWV unchanged at 6 months Femoral PWV decreased at 3 months (−22.8 ± 7.2%) Femoral PWV unchanged at 6 months NonG: Radial PWV unchanged at 3 months (+0.2 ± 4.2%) Radial PWV unchanged at 6 months Femoral PWV unchanged at 3 months (−0.7 ± 7.3%) Femoral PWV unchanged at 6 months
Madden et al. (35)	Healthy postmenopausal women 72 ± 1 years *n* = 26	12 weeks 3 times daily 10–20 min daily, 140 min/week Bench step exercise: Step height 15–20 cm; step rhythm initially 40 steps/min Level of LA and RPE: 6–20 Borg scale, end of exercise-LA > 4 mmol.L^-l^ and/or RPE > 17	AerG: baPWV decreased (−2.1 m/s) NonG: baPWV unchanged
Ohta et al. (36)	Male and female hemodialysis (HD) patients Median of 67 years *n* = 19	3 months 3 times per week Cycle ergometer Minimum 30 min	HD + AerG: PWV unchanged (10.4 ± 3.1 vs. 8.7 ± 2.7 m/s) HD only: PWV unchanged (9.8 ± 3.8 vs. 10.5 ± 3.6 m/s)
Toussaint et al. (37)	Hypertensive males and females 66.1 ± 4.0 years *n* = 24	12 weeks 3 times daily 20 min Arm-cycling Intensity at lactate concentrations of 2.0 ± 0.5 mmol.L^-l^	AerG: baPWV unchanged NonG: baPWV unchanged
Concurrent training			
Westhoff et al. (38)	Hypertensive postmenopausal women 70 ± 4 years *n* = 16	12 weeks 3 times daily 60 min *Circuit RT portion*: 4 exercises 4 sets Kettlebell mass of 2 kg *Circuit AER portion*: Step-box exercise 65–80% of age-predicted maximal HR	ConG: baPWV decreased (−0.7 m/s) NonG: baPWV unchanged
Jeon et al. (39)	7 older adult(s) women 68.8 ± 7.0 years *n* = 77	12 weeks 1 vs. 2 times per week 90 min *Circuit RT portion*: 6–8 exercises 3–5 sets Rubber tubes and dumbbells *AER portion*: Cycling for 20 min	1 day ConG: baPWV unchanged (−1.5 ± 7.5%) 2 day ConG: baPWV decreased (−7.8 ± 7.0%) NonG: baPWV unchanged (−0.4 ± 7.5%)
Miura et al. (40)	Hyper- and normotensive women Hypertensive 72.9 ± 5.7 years Normotensive 72.0 ± 7.1 years *n* = 284	12 weeks 2 times per week 90 min *Circuit RT portion*: 6–8 exercises 15–20 repetitions Rubber tube and/or lightweight dumbbells (500–1,000 g) *Circuit AER portion*: Walking or running for 10–15 m or cycling up to 20 min	Hypertensive ConG: baPWV decreased (1893.6 ± 304.5 vs. 1821.0 ± 311.8 cm/s; −72.5 ± 8.1 cm/s) Hypertensive NonG: baPWV unchanged (1830.8 ± 285.5 vs. 1841.9 ± 294.9 cm/s; +11.1 ± 72.4 cm/s) Normotensive ConG: baPWV decreased (1684.1 ± 200.2 vs. 1552.5 ± 208.6 cm/s; −131.5 ± 107.3 cm/s) Normotensive NonG: baPWV unchanged (1643.2 ± 211.4 vs. 1641.5 ± 203.6; −1.6 ± 84.3 cm/s)
Miura et al. (41)	Obese older men 68.8 ± 0.9 years *n* = 20	12 weeks 3 times per week *RT portion*: Elastic-band RT *AER portion*: Walking/running on a treadmill and bicycle at 60–70% of maximal heart rate	ConG: baPWV decreased NonG: baPWV unchanged
Park et al. (42)	Postmenopausal women 77 ± 2 years *n* = 101	52 weeks 3 times per week 60 min *RT portion*: 3 sets 10–15 repetitions RPE 12–14 *AER portion*: walking, jogging, cycling 50–60% HRR RPE 12–14	ConG: baPWV decreased (−0.7 m/s) NonG: baPWV unchanged
Pekas et al. (43)	Older men 70.5 ± 3.5 years *n* = 45	10 weeks 2 times per week *RT portion*: 5 exercises 3 sets 8–12 repetitions 70–80% of 1-RM *AER portion*: Cycling 60% of HRR	Aerobic before RT: cfPWV unchanged RT before aerobic training: cfPWV decreased (9.0 ± 1.6 vs. 8.0 ± 1.6 m/s) NonG: cfPWV unchanged
Shiotsu et al. (44)	Post-menopausal women 75 ± 2 years *n* = 20	12 weeks 3 times per week *RT portion*: Resistance bands 40–50% HRR in weeks 1–4; 60–70% HRR in weeks 9–12 *AER portion*: Walking 40–50% HRR in weeks 1–4; 60–70% HRR in weeks 9–12	ConG: baPWV decreased (−1.2 ± 0.4 m/s) NonG: baPWV unchanged

RT, resistance training; 1-RM, 1 repetition maximum; HRR, heart rate reserve; PWVao, aortic pulse wave velocity; HD, hemodialysis; RTG, resistance training group; NonG, non-exercising group; ConG, concurrent training group; cfPWV, carotid-femoral pulse wave velocity; baPWV, brachial-ankle pulse wave velocity; RPE, rating of perceived exertion; mmol.L^-l^, millimoles per litre.

To quantify the observed outcomes, the mean change in cfPWV for the three Resistance Training (RT) studies was negligible, ranging from −0.1 m/s to +0.1 m/s, confirming a lack of clinical effect. This finding of a neutral long-term effect from RT is consistent with major meta-analyses, such as those by Wainman (46) and Werner et al. (47), which found no significant change or only short-term increases in arterial stiffness following RT, particularly in older populations (61). In stark contrast, the single aerobic training (AER) study demonstrated a substantial PWV reduction of −1.0 m/s. This degree of benefit is supported by other systematic reviews, which generally report pooled PWV reductions from aerobic exercise in the range of −0.63 m/s to −1.29 m/s in comparable cohorts. This difference in magnitude underscores the superior effectiveness of aerobic training protocols over dynamic resistance training for improving arterial stiffness.

Across the studies reviewed, RT did not demonstrate a significant impact on arterial stiffness in older adults. This finding stands in contrast to cross-sectional studies involving younger populations, which suggest that RT, especially when conducted at high intensity, is associated with increased arterial stiffness (48, 49, 62). Importantly, the studies included here, which ranged in intensity from 70% 1-RM (30) to fatigue protocols (31, 32), did not demonstrate a deleterious effect on vascular function. These results align with previous findings showing that RT is not harmful to arterial compliance in older adults (50). However, the predominance of mixed-sex samples limits conclusions about potential sex-specific responses.

Whilst resistance training may not affect arterial stiffness, it remains a crucial part of exercise prescriptions for older adults due to its unique health benefits. Resistance training is essential for counteracting sarcopenia by promoting muscle growth, enhancing muscular power, and decreasing the risk of falls (51). Since arterial stiffening is associated with skeletal muscle mass decline (2), “hypertrophic” RT indirectly benefit vascular health. Future research should explore novel RT modalities (i.e., with varying volumes, loads, velocities, etc.) that enhance muscle outcomes whilst minimising health hazards to the vasculature (52). High-velocity concentric resistance training, for example, may have a smaller impact on arterial stiffness due to a reduced vasopressor response (52). Whilst careful selection of exercises could also limit stiffening associated with large muscle groups and upper-body eccentric movements (25).

Most studies reported a destiffening effect of aerobic training in older adults (33–37). However, findings were inconsistent in specific populations, such as hemodialysis patients (36) and older adult(s) hypertensives (37). One possible explanation is exercise modality: for instance, arm cycling, used by Westhoff et al. (38), engages a smaller muscle mass and provokes a greater vasopressor response compared to lower-body aerobic exercise (53). These methodological differences may explain variability in outcomes. Sex-specific remains underexplored, with only one study (36) addressing this question directly. Given known biological differences in vascular ageing, future studies should prioritise sex-stratified analyses.

Evidence from previous research strongly supports concurrent aerobic and resistance training as the most effective approach for reducing arterial stiffness in the older adult(s). Notably, Shiotsu et al. (44) found that performing RT before aerobic training produced a greater reduction in pulse wave velocity (PWV) than the reverse sequence (43). Similar benefits have also been observed in younger adults (25). These findings underscore the potential of concurrent training to not only mitigate any neutral or adverse effects of RT but also to combine the unique benefits of both modalities. Beyond vascular outcomes, concurrent training improves multiple cardiovascular risk factors, functional capacity, quality of life, and even psychological well-being (54), making it a particularly relevant strategy for both prevention and rehabilitation in ageing populations.

The differential effects of training modalities likely reflect distinct physiological mechanisms. Aerobic training reduces sympathetic nervous system activity, lowers vascular resistance, and enhances arterial compliance (21). resistance training may contribute by increasing muscle strength, which can indirectly improve arterial stiffness through better blood pressure regulation (22). Mechanistically, it increases phosphorylation of endothelial nitric oxide synthase (eNOS), improves nitric oxide bioavailability, and reduces inflammation (26, 55). By contrast, RT may indirectly support vascular health through blood pressure regulation and improvements in muscle mass and strength (22). However, high-intensity RT has been linked to increased endothelin-1, a potent vasoconstrictor that may offset eNOS activation (26). These findings highlight the need for mechanistic studies to clarify how RT, when combined with aerobic training, produces synergistic benefits for arterial stiffness. Rakobowchuk et al. (26) reported that the RT group’s PWV was unchanged (+1.04 m/s), whilst Guilherme Silva de Mendonça et al. (12) found that aortic PWV (PWVao) was unchanged (10.34 vs. 10.54 ± 2 m/s).

This review has both strengths and limitations. A notable strength is its focus on an underexplored population, as most previous studies on exercise and arterial stiffness have primarily involved younger adults (23, 56). By including older participants with diverse health conditions, the findings of this review are more clinically relevant. The outcomes were rated as moderate-certainty evidence, giving confidence in the estimates of effect. The observed non-significant PWV change must be interpreted considering our cohort’s composition, which was predominantly male and of Caucasian men origin.

However, several limitations need to be acknowledged, many of which are inherent to the nature of a systematic review. As a retrospective methodology, this review is bounded by the quality and design of the primary studies. Specifically, the wide variation (heterogeneity) in study designs, participant populations, exercise protocols, and arterial stiffness outcome measures (e.g., cfPWV vs. baPWV) made direct statistical comparisons challenging, necessitating a narrative approach to data synthesis. Moreover, any limitations or biases present in the original research could be carried forward and potentially amplified in this synthesis. For example, in some cases, details about the interventions were insufficiently described, hindering a complete evaluation of the training characteristics. Additionally, methods for assessing arterial stiffness varied (e.g., PWV versus augmentation index).

This review also highlights the implications of RT on vascular health and provides suggestions for future research. Whilst RT alone does not significantly impact arterial stiffness, combining it with aerobic training maximises vascular benefits and yields important improvements in musculoskeletal health and functional capacity. Future studies should focus on optimising concurrent training protocols by systematically adjusting key variables such as intensity, volume, and exercise order. Randomised controlled trials, particularly those including non-exercising control groups, are crucial for establishing the causal effects of these interventions. Additionally, future research should incorporate sex-specific analyses to determine whether vascular adaptations differ between older men and women. A better understanding of these factors will enable the development of more tailored and effective exercise prescriptions aimed at improving vascular health and overall well-being in older adults. Future studies should focus on optimising concurrent training protocols by systematically adjusting key variables such as intensity, volume, and exercise order. Furthermore, a critical area for future research involves conducting comparative studies between older and younger populations to clearly delineate age-related differences in vascular adaptations and further reinforce the clinical relevance of these findings in geriatric care. Finally, restricting the review to English-language studies may have introduced a language bias, a form of selection bias, by excluding potentially relevant research from non-English sources. Despite employing a rigorous, multi-database search strategy, the potential for residual publication bias, where studies with non-significant findings are less likely to be published, cannot be entirely eliminated.

## 5 Conclusion

RT is essential for maintaining strength and muscle mass in older adults. However, it does not significantly improve arterial stiffness. Importantly, RT does not negatively impact vascular function, even at higher intensities. In contrast, aerobic training consistently improves arterial stiffness. When combined with RT in a concurrent program, the benefits for vascular health are maximised. The findings also suggest that performing aerobic activity before RT and prioritising longer durations of aerobic exercise may further enhance vascular outcomes. Overall, these findings reveal that a well-planned concurrent exercise training program emerges as the most effective strategy for enhancing both cardiovascular and musculoskeletal health. This approach offers invaluable insights for health promotion and clinical rehabilitation, particularly for older adult(s) individuals facing an increased risk of cardiovascular disease. Additionally, future research should incorporate sex-specific analyses to determine whether vascular adaptations differ between older men and women. A better understanding of these factors will enable the development of more tailored and effective exercise prescriptions aimed at improving vascular health and overall well-being in older adults.

## Data Availability

The raw data supporting the conclusions of this article will be made available by the authors, without undue reservation.
